# The Transplant Evaluation Rating Scale Predicts Clinical Outcomes 1 Year After Lung Transplantation: A Prospective Longitudinal Study

**DOI:** 10.3389/fpsyt.2021.704319

**Published:** 2021-08-26

**Authors:** Mariel Nöhre, Martina de Zwaan, Maximilian Bauer-Hohmann, Fabio Ius, Christina Valtin, Jens Gottlieb

**Affiliations:** ^1^Department of Psychosomatic Medicine and Psychotherapy, Hannover Medical School, Hannover, Germany; ^2^Member of the German Center for Lung Research (DZL), Biomedical Research in End-stage and Obstructive Lung Disease Hannover, Hannover, Germany; ^3^Department of Cardiac, Thoracic, Transplant, and Vascular Surgery, Hannover Medical School, Hannover, Germany; ^4^Department of Respiratory Medicine, Hannover Medical School, Hannover, Germany

**Keywords:** lung transplantation, Transplant Evaluation Rating Scale, psychosocial functioning, quality of life, psychosomatic medicine

## Abstract

**Objectives:** It has been recommended that all candidates for lung transplantation undergo pre-transplant psychosocial evaluation for risk assessment. However, psychosocial issues are only important if they correlate with outcomes after transplantation.

**Methods:** In this prospective study patients who were referred for lung transplantation from 2016 to 2018 (*n* = 352) at Hannover Medical School were evaluated using the Transplant Evaluation Rating Scale (TERS). Clinical outcomes included listing, and post-transplant outcomes including mortality, medical aspects such as lung allograft dysfunction, hospitalizations, and renal function, behavioral aspects such as BMI and adherence, and mental issues such as levels of depression, anxiety, and quality of life. TERS scores were divided into tertiles and, in addition, the impact of the two subscale scores—“defiance” and “emotional sensitivity”—was investigated.

**Results:** Of the patients who were transplanted (*n* = 271) and were still alive (*n* = 251), 240 had already reached their 1-year assessment at the end of 2020 and were evaluated 1 year after the operation. A subgroup of 143 received an extended mental assessment. BMI, adherence scores, levels of anxiety, depression, and quality of life 1 year post-transplantation differed significantly between TERS tertiles with higher TERS scores predicting less favorable outcomes. The TERS subscale “defiance” was predictive of BMI and adherence whereas the TERS subscale “emotional sensitivity” was predictive of symptoms of anxiety and depression, and quality of life 1 year after transplantation. Patients in the lowest TERS tertile were more likely to having been listed and—as a trend—to having survived the first year after transplantation

**Conclusions:** Our findings show that psychosocial factors as measured by TERS score are predictors of behavioral and mental outcomes 1 year after lung transplantation. The TERS allows us to focus on psychosocial risk factors that can be treated or minimized before or after transplantation.

## Introduction

Lung transplantation is an accepted treatment option for patients with irreversible chronic lung disease, with more than 4,500 procedures performed per year worldwide and 350 in Germany (1, 2). In patients awaiting lung transplantation, symptoms of depression and anxiety and poor pre-transplant quality of life are highly prevalent (3–5) and may be associated with worse post-transplant outcomes, including increased mortality (6, 7). All patients who are considered lung transplant candidates usually undergo a transplant evaluation that includes both medical and psychosocial aspects. The objective of pre-surgical psychosocial evaluations is to identify patients at risk for medical, behavioral and emotional complications during and after organ transplantation. Thus, this evaluation is supposed to judge suitability for transplantation and to guide proactive interventions before and after transplantation (8).

In patients after lung transplantation, the literature on psychosocial predictors on a wider range of outcome measures is relatively scarce. One study did not find a predictive value of the Psychosocial Assessment of Candidates for Transplant (PACT) on 1-year survival (9) while a more recent study found a significant predictive value of this instrument on longer-term (12 year) survival in lung transplant recipients (10). Others found an association between specific pre-transplant psychosocial factors (executive functioning, memory performance, quality of well-being) and mortality following lung transplantation (11, 12). However, these studies did not use a structured psychosocial risk scale that accounts for all psychosocial factors.

The guideline of the German Medical Association concerning lung transplantation dictates that lung transplant candidates should be evaluated by a mental health professional before transplantation (13). Currently, the TERS is the most frequently used instrument (14, 15). The TERS has demonstrated efficacy in predicting peri- and post-transplant outcomes in patients receiving heart, lung, and liver transplants, but also bone marrow or stem cell transplantation as well as left ventricular assist device implantation (15–20). In previous studies of our group in patients prior to lung transplantation, we evaluated the level of psychosocial functioning using the TERS and validated the TERS and its subscales specifically in patients awaiting lung transplantation (21). However, we did not perform detailed follow-up analyses.

Also, even though survival is by far the most relevant outcome, it is not the only outcome. In addition to survival and transplant rates, success in lung transplantation should also be defined by patient-centered outcomes such as levels of depression and quality of life (22–24). For the evaluation of treatment effectiveness quality of life has become a meaningful clinical endpoint (25). This recognizes that the perspectives of patients are unique and may differ from those of clinicians. Additionally, prediction of adherence is crucial because non- or hypo-adherence to immunosuppressive medication and necessary medical recommendations is closely associated with a less favorable outcome also after lung transplantation (26, 27).

Up to now, no prospective studies have examined the predictive value of TERS scores with regard to a large number of peri- and 1-year post-transplant outcomes in patients awaiting lung transplantation. This would potentially drive attention to and help address specific needs in patients with specific characteristics. Thus, the objective of this single-institution study was to assess the impact of psychosocial factors as measured by TERS score on medical, behavioral, and psychosocial outcomes 1 year after lung transplantation. More specifically, in our study peri- and post-transplant outcomes included listing, mortality, prevalence of chronic lung allograft dysfunction, hospitalizations, renal function, weight, adherence, levels of depression and anxiety, and quality of life. In line with the results in the literature, we expected higher pre-transplantation TERS scores to be associated with poorer medical outcomes, poorer adherence, higher levels of depression and anxiety, and lower quality of life 1 year post-transplantation.

## Methods

### Patients and Procedures

Trained residents and master-level psychologists conducted a TERS interview according to a structured protocol during routine psychosocial clinical assessment prior to enlistment for lung transplantation. The structured protocol contains the modules for affective disorders, anxiety disorders, adjustment disorders, substance use disorders, and somatoform disorders of the Structured Clinical Interview for DSM IV disorders (28). Patients (*n* = 352) presenting for psychosocial evaluation prior to lung transplantation in 2016, 2017, and 2018 participated. All lung transplant recipients received scheduled follow-up care at the transplant center. The ethics committee of Hannover Medical School approved the study and all participants gave written informed consent before study entry.

### Instruments

#### Pre-transplantation

The Transplant Evaluation Rating Scale (TERS) (13, 14) is an expert interview for the assessment of psychosocial functioning prior to organ transplantation with satisfying inter-rater reliability scores (kappa between 0.8 and 0.9) (29). The German version has been validated in patients awaiting lung transplantation (21). It covers 10 distinct domains of psychosocial functioning considered relevant for adjustment to transplantation and its consequences: (a) current or past mental disorders (axis 1 according to DSM-IV), (b) personality disorders (axis 2 according to DSM-IV), (c) substance use/abuse, (d) compliance, (e) health behaviors, (f) quality of family and social support, (g) history of coping, (h) current coping with disease and treatment, (i) quality of affect and, (j) mental/cognitive status (past and present). Each of the 10 domains is rated by a clinician on a three-point scale based on the level of presence of symptoms within each domain (1 = minimal/mild, 2 = moderate, 3 = severe impairment). Reflecting the importance of the respective domain for the overall level of psychosocial functioning, each item rating is multiplied by a priori assigned weight (ranging from 1 to 4) and the items are added up to calculate the total (weighted) score (range 26.5-79.5). Higher scores represent greater impairment in the levels of psychosocial functioning. Several research groups have detected a two-factor structure of the TERS in different transplant sample named “defiance” and “emotional sensitivity” which showed differential convergent and predictive validity (21). “Defiance” is a clearly demarcated behavioral factor comprised of a history of difficulties with substance abuse/use, health self-care, non-compliance, family support, personality disorders, and general coping. “Emotional sensitivity” is composed of items tapping quality of affect, adjustment to illness, mental status, and mental disorders. On the basis of a patient's weighted total score, patients were divided into three tertile groups. The tertile method has been recommended since it does not cause inflation of *p*-values compared with outcome dependent cut points (17, 19). For the two subscales we used the median as a cutoff. Even though the TERS was not developed as a scalable instrument we calculated Cronbach's alpha (α = 0.647).

Other clinical variables included demographic information and pulmonary diagnosis. Patients were asked to report their age, sex, years of completed education, and partnership status. Patients were classified into four categories depending on their underlying disease (2): category A, obstructive airway diseases (e.g., chronic obstructive pulmonary disease [COPD]); category B, diseases of the pulmonary circulation (e.g., idiopathic pulmonary arterial hypertension); category C, infectious lung diseases (e.g., cystic fibrosis [CF]); and category D, restrictive lung diseases (e.g., pulmonary fibrosis).

#### Post-transplantation

##### Medical Outcomes

Enlistment, patient survival, prevalence of chronic lung allograft dysfunction (FEV1 <80%), number and duration of hospitalizations, renal function (eGFR), and overall comorbidity (Charlson Comorbidity Index, CCI) (30) were taken from our comprehensive institutional database.

##### Behavioral Outcomes

To assess adherence, five domains were evaluated using a three-level Likert scale (26) with an overall adherence rating between 0 and 100%. The five domains include: (1) health perception (e.g., inconsistent medication knowledge, tobacco/drug abuse, poor diabetic control, use of sunbeds), (2) home spirometry frequency, (3) contact (e.g., missed appointments), (4) nutrition, exercise (e.g., regular exercise, normal-weight), and (5) trough levels in target range. Adherence ratings were completed at each post-operative visit. Scores were assigned by transplant coordinators and discussed with physicians during daily team meetings. The mental health professional was not involved in the rating of the five adherence domains. Mean adherence scores including all available post-operative ratings up to 1 year were calculated. In a recent study from our center including patients from 2010 to 2013 the median adherence score was 86% in the first 3 years after transplantation. After 5 years, patients below and above this cutoff differed significantly with regard to allograft and patient survival and chronic allograft dysfunction (26). Thus, we used the cutoff of 86% to differentiate between good and suboptimal adherence in our sample.

To estimate the immunosuppressive drug adherence we used the four-item interview version of the Basel Assessment of Adherence to Immunosuppressive Medication Scale (BAASIS^©^) (31). Participants were asked about how often, over the last 4 weeks, they (1) had not taken their drugs (taking dimension), (2) had taken their medication more than 2 h before or after their prescribed taking time (timing dimension), (3) had skipped at least two consecutive doses of their drugs (drug holidays), and/or (4) had reduced the prescribed amount of their medication (dose reduction). Responses were given on a six-point scale ranging from 0 (never) to 5 (every day). Non-adherence was dichotomously defined as any self-reported non-adherence on any of the four items.

##### Psychological Outcomes

*Depression and Anxiety*. All patients filled out the four-item Patient Health Questionnaire (PHQ-4) (32), an ultra-brief self-report questionnaire that consists of a two-item depression scale (PHQ-2) and a two-item anxiety scale (GAD-2). Replies are rated on a four-point Likert scale (0 = not at all to 3 = nearly every day). Thus, the total score of the scale ranges between 0 and 12 points. In the current study, the Cronbach's α for the overall score was 0.842. PHQ-4 scores of 6 or above are considered indicative for the presence of a depressive or anxiety disorder. For the PHQ-2 and the GAD-2, scale scores of ≥3 were suggested as cut-off points between the normal range and probable cases of depression or anxiety, respectively.

The subgroup of 143 patients who participated in a more detailed psychosocial assessment also completed the Patient Health Questionnaire-9 (PHQ-9) (33, 34), a self-report instrument screening for symptoms of depression over the last 2 weeks. Nine items are rated on a four-point Likert scale (0 = not at all to 3 = nearly every day) (Cronbach's α = 0.811). They also completed the Generalized Anxiety Disorder-7 (GAD-7) (35, 36), a self-report instrument screening for symptoms of generalized anxiety during the last 2 weeks. Seven items are rated on a four-point Likert scale (0 = not at all to 3 = nearly every day) (Cronbach's α = 0.895). In both scales, all scores are summed up into a total score, with higher scores representing higher levels of depressive and anxiety symptoms, respectively. For both scale values from 5 to 9 represent mild, from 10 to 14 moderate, and ≥15 severe symptom severity.

*Quality of Life*. Self-rated levels of Quality of Life (QoL) were assessed during the clinical interviews with a visual analog scale by asking patients: “on a scale of 0-10, with 10 meaning perfectly satisfied, how satisfied are you with your current quality of life?” (QoL VAS).

The subgroup of 143 patients also completed the Pulmonary-specific Quality-of-Life Scale (PQLS), a self-report questionnaire assessing quality of life specifically in patients with end-stage lung diseases (25, 37). The scale consists of 25 items which are rated on a five-point-Likert-scale ranging from 1 (“not at all”) to 5 (“most of the time”). A total score between 25 and 125 can be reached with higher values indicating lower quality of life (Cronbach's α = 0.871). Three subscales (“task interference,” “psychological,” and “physical”) were identified in the original English version of the PQLS (25). The subscale “task interference” (eight items) (Cronbach's α = 0.801) focuses on occupational and social functioning, the subscale “psychological” (seven items) (Cronbach's α = 0.833) assesses mental and psychological aspects, and the subscale “physical” (four items) (Cronbach's α = 0.884) evaluates physical functioning. Six items do not load on any factors; thus, the total scale is also reported.

They also completed the SF-8, a short form of the SF-36 Health Survey, which is used for generic assessment of physical and mental aspects of health-related quality of life (HRQoL) (38) In the SF-8 each of the 8 SF-36 dimensions is represented by a single item to be assessed over the last 4 weeks (Cronbach's α = 0.867). The values of these eight dimensions were aggregated to a physical component summary (PCS) value and a mental component summary (MCS) value which were converted to a standardized T score. The T score is a metric with a mean of 50 and standard deviation of 10 that has been normalized to the US general population. German reference values are available, allowing a comparison between the T scores of our sample and German norms (39).

### Statistical Analyses

Statistical analyses were performed using SPSS (IBM SPSS Statistics for Windows, Version 25.0. Armonk, NY: IBM Corp.). Categorical variables are presented as numbers (n) and percentages (%), continuous variables as median and range. Post-transplant outcomes were compared between the TERS tertile groups and between the median split subscale scores “defiance” and “emotional sensitivity” using Kruskal-Wallis *H*-tests and Mann-Whitney *U*-tests for continuous variables and chi-square tests for categorical variables. In addition to significance testing, we calculated Cramer V as effect size (40) for chi-square tests: 0.1 indicates a small effect, 0.3 a medium effect, and 0.5 a large effect and eta squared (η^2^) as effect size for non-parametric tests: 0.01 indicates a small effect, 0.06 a medium, and 0.14 a large effect. Binary logistic regression analyses were conducted with significant outcomes as the dependent variable (adherence, BMI, PHQ-4) and TERS tertiles and the two subscale scores, respectively, as the main independent variable controlling for the baseline variables age, sex, educational level, and pulmonary diagnosis. The level of significance was set at ≤ 0.05.

## Results

### Sample

**Figure 1:**
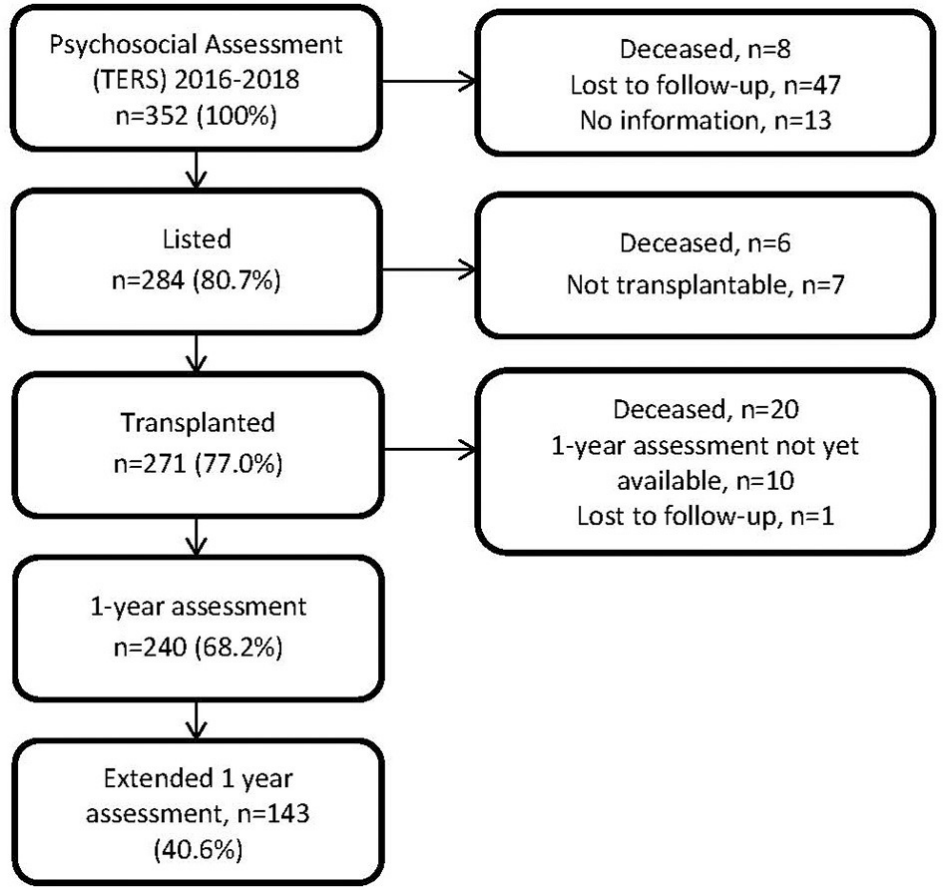
Flowchart of participating patients.

Of the patients who were transplanted (*n* = 271) and were still alive (*n* = 251), 240 had already reached their 1-year assessment at the end of 2020 and were evaluated (Figure 1). Overall, 34 patients had died, 14 before transplantation and 20 (7.4%) of transplanted patients during the first year after transplantation. The median age of our patient sample 1 year after transplantation (*n* = 240) was 55.7 years (range 20-71), 114 (47.5%) were women (Table 1). Most patients underwent bilateral lung transplantation (*n* = 237), 10 patients underwent single lung transplantation, 1 patient combined heart-lung transplantation, 2 patients combined lung-liver transplantation, and 21 patients had a double lung re-transplantation due to bronchiolitis obliterans syndrome (BOS). The median LAS score was 34.5 (range 30.6-77.9) with 12 (5%) patients reaching a final pre-transplant lung allocation score (LAS) of 50 or above, which is considered “high” (2). Seventy-two (30%) of the patients met criteria for a lifetime mental disorder and 46 (19.2%) for a current mental disorder. Seventy patients (29.2%) reported experience with psychological/psychiatric treatments and 49 (20.4%) with psychopharmacological treatment. The most frequent diagnoses were affective and anxiety disorders. Sixty patients (25%) had the minimal score on the TERS of 26.5 and 18 (7.5%) scored in the high risk group (≥37.5) as defined by Hoodin and Kalbfleisch (20). The total population was stratified according to their TERS scores into tertiles. The three TERS tertiles did not differ with regard to age, sex, and partnership status; however, patients in the highest tertile were significantly less educated and were more often diagnosed with an obstructive lung disease (Table 2). These differences were mainly due to differences in the “defiance” subscale (Table 3).

**Table 1:** Baseline characteristics of different samples.

	**TERS pre tx available** ***N* = 352**	**Listed** ***N* = 284**	**Transplanted** ***N* = 271**	**1-year post tx assessment** ***N* = 240**	**Extended 1-year post tx assessment,** ***N* = 143**
Sex, *n* (%)					
Female	165 (46.9)	134 (47.2)	129 (47.6)	114 (47.5)	66 (46.2)
Male	187 (53.1)	150 (52.8)	142 (52.4)	126 (52.1)	77 (53.8)
Age at TERS assessment, median (range)	53.3 (18-70)	53.5 (18-70)	54 (18-70)	54.3 (18-70)	54.5 (18-70)
Educational level, *n* (%)	*N* = 350	*N* = 283	*N* = 270	*N* = 239	*N* = 142
<12 years	248 (70.9)	197 (69.6)	187 (69)	165 (69)	99 (69.7)
≥ 12 years	102 (29.1)	86 (30.4)	83 (31)	74 (31)	43 (30.3)
Partnership, *n* (%)					
Yes	280 (79.5)	237 (83.5)	225 (83)	201 (83.8)	120 (83.9)
No	72 (20.5)	47 (16.5)	46 (17)	39 (16.3)	23 (16.1)
LAS category, *n* (%)					
Category A	—	91 (32)	89 (32.8)	79 (32.9)	45 (31.5)
Category B	—	18 (6.3)	16 (5.9)	14 (5.8)	7 (4.9)
Category C	—	55 (19.4)	50 (18.5)	45 (18.8)	35 (24.5)
Category D	—	120 (42.3)	116 (42.8)	102 (42.5)	56 (39.2)
Last pre tx LAS score, median (range)			*N* = 270		
	—	—	34.5 (30.6-94.2)	34.5 (30.6-77.9)	34.5 (30.6-77.9)
Pre tx BMI, kg/m^2^, median (range)	*N* = 347	*N* = 263	*N* = 263		
	22.0 (14-34.3)	22.9 (14.1-32.5)	22.4 (14-34.3)	22.4 (14-32.5)	22.5 (14-32.5)
TERS weighted score, median (range)	30.5 (26.5-57.0)	30.8 (26.5-48.5)	30.0 (26.4-48.5)	30.0 (26.5-48.5)	30.5 (26.5-48.5)

*BMI, Body Mass Index; LAS Category, pulmonary diagnosis according to the Lung Allocation Score (Category A, obstructive airway diseases; category B, diseases of the pulmonary circulation, category C, infectious lung diseases, category D, restrictive lung diseases); TERS, Transplant Evaluation Rating Scale; pre tx, pre-transplantation*.

**Table 2:** Comparison of baseline characteristics divided by TERS tertiles.

**Variable**	***N***	**TERS ≤ 28**	**TERS 29-31.5**	**TERS ≥ 32**	****χ^2^ or *H*, *p*-value****
		***N* = 80**	***N* = 81**	***N* = 79**	
**TERS scores**, ***n*****(%) or median (range)**					
Age at 1 year post tx, median (range)	240	53 (21-71)	54 (20-66)	57 (22-67)	*H* = 2.742, *p* = 0.254
Sex, *n* (%)	240				
female	114	35 (43.8)	39 (48.1)	40 (50.6)	χ^2^ = 0.776 (df = 2), *p* = 0.679
male	126	45 (56.3)	42 (51.9)	39 (49.4)	
Educational level, *n* (%)	239				
<12 years	165	44 (55.0)	57 (71.3)	64 (81.0)	χ^2^ = 12.858 (df = 2), *p* = 0.002
≥12 years	74	36 (45.0)	23 (28.7)	15 (19.0)	
Partnership, *n* (%)	240				
yes	201	66 (82.5)	68 (84.0)	67 (84.8)	χ^2^ = 0.159 (df = 2), *p* = 0.923
No	39	14 (17.5)	13 (16.0)	12 (15.2)	
LAS-Category, *n* (%)	240				
Category A	79	10 (12.5)	34 (42.0)	35 (44.3)	χ^2^ = 27.436 (df = 6), *p* <0.001
Category B	14	4 (5.0)	5 (6.2)	5 (6.3)	
Category C	45	20 (25.0)	17 (21.0)	8 (10.1)	
Category D	102	46 (57.5)	25 (30.9)	31 (39.2)	

*Univariate analyses (Kruskal-Wallis H-tests, Chi square tests).*

*LAS Category, pulmonary diagnosis according to the Lung Allocation Score (Category A, obstructive airway diseases; category B, diseases of the pulmonary circulation, category C, infectious lung diseases, category D, restrictive lung diseases); TERS, Transplant Evaluation Rating Scale; post tx, post-transplantation*.

**Table 3:** Comparison of baseline characteristics divided by the TERS subscales “defiance” and “emotional sensitivity” (median split).

**Variable**	***N***	**Defiance** **≤ 18.75** ***N* = 120**	**Defiance** **> 18.75** ***N* = 120**	**χ^2^** **or Z, *p*-value,** **effect size**	**Emotional** **≤ 10** ***N* =132**	**Emotional** **> 10** ***N* = 108**	**χ^2^ or *Z***, ***p*-value, effect size**
**TERS subscales median split;** ***n*** **(%) or median (range)**							
Age	240	51 (20-71)	58 (22-67)	*Z* = −3.325, *p* = 0.001	55 (21-71)	56 (20-67)	*Z* = −0.481, *p* = 0.630
Sex	240						
female		58 (48.3)	56 (46.7)	χ^2^ = 0.067 (df = 1), *p* = 0.796	58 (43.9)	56 (51.9)	1.491 (df = 1), *p* = 0.222
male		62 (51.7)	64 (52.5)		74 (56.1)	52 (48.1)	
Educational level	239						
<12 years	165	66 (55.5)	99 (82.5)	χ^2^ = 20.434 (df = 1), *p* <0.001, *V* = 0.292	88 (66.7)	77 (72.0)	χ^2^ = 0.775 (df = 1); *p* = 0.379
≥12 years	74	53 (44.5)	21 (17.5)		44 (33.3)	30 (28.0)	
LAS-Category, *n* (%)	240						
Category A	79	18 (15)	61 (50.8)	χ^2^ = 41.812 (df = 3), *p* <0.001, *V* = 0.417	39 (29.5)	40 (37.0)	χ^2^ = 2.154 (df = 3), *p* = 0.541
Category B	14	8 (6.7)	6 (5.0)		9 (6.8)	5 (4.6)	
Category C	45	36 (30.0)	9 (7.5)		24 (18.2)	21 (19.4)	
Category D	102	58 (48.3)	44 (36.7)		60 (45.5)	42 (38.9)	

*Univariate analyses (Mann-Whitney tests, Chi square tests).*

*LAS Category, pulmonary diagnosis according to the Lung Allocation Score (Category A, obstructive airway diseases; category B, diseases of the pulmonary circulation, category C, infectious lung diseases, category D, restrictive lung diseases); TERS, Transplant Evaluation Rating Scale*.

Standard maintenance immunosuppression consisted of a triple drug regimen including a calcineurin inhibitor (CNI), prednisolone and mycophenolate mofetil.

Baseline characteristics of all patients who received the TERS (*n* = 352), of the listed patients (*n* = 284), of the transplanted patients (*n* = 271), of the patients with 1-year assessments (*n* = 240), and of patients who participated in the extended 1-year assessment (*n* = 143) are summarized in Table 1. No major differences between samples could be detected.

### Prediction of Outcome

#### Medical Outcomes

As of 31th December 2020, 284 patients of the entire sample of 352 psychologically assessed patients had been listed (80.7%). The percentage of patients listed in the low, intermediate and high TERS tertile were 86.1, 82.1, and 72.4% which was significantly different [χ^2^ = 8.131 (df = 2) *p* = 0.017; Cramer-V = 0.152]. Of those who died during the first year after transplantation (*n* = 20), 15% were in the low, 60% in the intermediate, and 25% in the high TERS tertile. This difference approached statistical significance with a small effect size [χ^2^ = 5.858 (df = 2) *p* = 0.053; Cramer-V = 0.150].

One-year renal function (eGFR), forced expiratory volume in 1 s (FEV1) <80% in relation to the post-transplant baseline FEV1, number of hospitalizations during the first year, and the CCI were not different between TERS groups (Table 2). This was also true for the two TERS subscales (data not shown).

#### Behavioral Outcomes

Overall, 5% (*n* = 12) of the patients were obese (BMI ≥ 30 kg/m^2^), 33.2% (*n* = 79) were overweight and 6.7% (*n* = 16) were underweight 1 year after transplantation. Most patients were in the normal-weight range (55%, *n* = 131). Patients in the higher TERS tertiles were more often obese (11.4%) and overweight (39.2%) (Table 4).

**Table 4:** Comparison of 1-year post-transplant outcomes divided by TERS tertiles (*n* = 240).

**Variable**	***N***	**TERS ≤ 28** ***N* = 80**	**TERS 29-31.5** ***N* = 81**	**TERS ≥ 32** ***N* = 79**	**χ^2^ or *H*, *p*-value,** **effect size**
**TERS scores**, ***n*****(%) or median (range)**					
BMI kg/m^2^, *n* (%)	238				
<18.5 (underweight)	16	7 (8.9)	4 (5.0)	5 (6.3)	χ^2^ = 15.958 (df = 6), *p* = 0.014, V = 0.183
18.5-24.9	131	49 (62.0)	48 (60.0)	34 (43.0)	
25-29.9 (overweight)	79	23 (29.1)	25 (31.1)	31 (39.2)	
≥30 (obesity)	12	0 (0)	3 (3.8)	9 (11.4)	
eGFR quartiles	240				
≤ 38.5	60	17 (21.3)	17 (21.0)	26 (32.9)	χ^2^ = 7.798 (df = 6), *p* = 0.253
38.6-54.5	60	18 (22.5)	25 (30.9)	17 (21.5)	
54.6-68.9	60	19 (23.8)	20 (24.7)	21 (26.6)	
≥69	60	26 (32.5)	19 (23.5)	15 (19.0)	
CCI	240				
CCI = 0	144	49 (61.3)	48 (59.3)	47 (59.5)	χ^2^ = 0.079 (df = 2), *p* = 0.961
CCI > 0	96	31 (38.8)	33 (40.7)	32 (40.5)	
No. of hospitalizations during first year after tx	240				
0	115	39 (48.8)	40 (49.4)	36 (45.6)	χ^2^ = 0.266 (df = 2), *p* = 0.875
≥1	125	41 (51.2)	41 (50.6)	43 (54.4)	
FEV1 %	240				
<80%	99	37 (46.3)	33 (40.7)	29 (36.7)	χ^2^ = 1.506 (df = 2) *p* = 0.471
≥80%	141	43 (53.8)	48 (59.3)	50 (63.3)	
Adherence score (mean during first year after tx)	240				
<87%	110	31 (38.8)	33 (40.7)	46 (58.2)	χ^2^ = 7.351 (df = 2), *p* = 0.025, *V* = 0.175
≥87%	130	49 (61.3)	48 (59.3)	33 (41.8)	
Health perception	240	0 (0-1)	0.1 (0-1)	0.3 (0-1.3)	*H* = 14.936, *p* =0.001 η^2^ = 0.055
Home spirometry frequency	240	0 (0-1.4)	0 (0-1)	0 (0-1.7)	*H* = 3.261, *p* = 0.196
Contact	240	0 (0-0.6)	0 (0-0.7)	0 (0-0.9)	*H* = 4.439, *p* = 0.109
Nutrition, exercise	240	0.3 (0-1.7)	0.2 (0-1.4)	0.4 (0-1.7)	*H* = 5.019, *p* = 0.081
Trough levels	240	0.6 (0-1.3)	0.6 (0-1.5)	0.6 (0-2)	*H* = 0.94, *p* = 0.625
Total score	240	1.2 (0-4.6)	1.2 (0-4.3)	1.4 (0.2-5.7)	*H* = 3.865, *p* = 0.145
Percentage	240	88.5 (53.8-100)	88.3 (56.7-100)	86 (43.3-98.3)	*H* = 3.865, *p* = 0.145
QoL VAS, median (range)	235	8 (3.5-10)	8.5 (1-10)	8 (2-10)	*H* = 0.224, *p* = 0.894
PHQ-4 total, median (range)	235	0 (0-5)	0 (0-8)	0 (0-12)	*H* = 3.376, *p* = 0.185
PHQ 4 median split	235				
<1	143	54 (68.4)	49 (62.0)	40 (51.9)	χ^2^ = 4.475 (df = 2), *p* = 0.107
≥1	92	25 (31.6)	30 (38.0)	37 (48.1)	

*Univariate analyses (Kruskal-Wallis H-tests, Chi square tests).*

*BMI, Body Mass Index; CCI, Charlson Comorbidity Index; eGFR, estimated glomerular filtration rate; FEV1, forced expiratory volume in 1 s (% of first post tx value); PHQ-4, Patient Health Questionnaire-ultrashort version; QoL VAS, quality of life visual analog scale; TERS, Transplant Evaluation Rating Scale; tx, transplantation*.

Overall, 45.8% exhibited an adherence score of <87% indicating suboptimal adherence to components of the medical regimen and transplant program recommendations. More patients in the highest TERS tertile were rated with an adherence score of <87% (58.2%) (Table 4).

Both associations (TERS with BMI and adherence, respectively) were mainly due to differences in the “defiance” subscale categories and not the “emotional sensitivity” subscale categories (Table 5). Logistic regression analysis adjusted for baseline variables confirmed these significant associations (Supplementary Tables 1, 2). Looking at the individual components of non-adherence, low health perception (e.g., inconsistent medication knowledge, recommendations regarding substance abuse not met, poor diabetic control, use of sunbeds) and missed appointments with the transplant center were predicted by the TERS but not home spirometry frequency, nutrition and exercise, or trough levels outside the target range.

**Table 5:** Comparison of 1-year follow-up outcomes divided by the TERS subscales “defiance” and “emotional sensitivity” (median split).

**Variable**	***N***	**Defiance** **≤ 18.75,** ***N* = 120**	**Defiance** **> 18.75,** ***N* = 120**	****χ^2^ or *Z*, *p*-value, effect size****	**Emotional** **≤ 10,** ***N* = 132**	**Emotional > 10,** ***N* = 108**	****χ^2^ or *Z*, *p*-value, effect size****
**TERS subscales median split;** ***n*** **(%) or median (range)**							
BMI kg/m^2^, *n* (%)	238						
<18.5	16	9 (7.6)	7 (5.9)	*X*^2^ = 17.013 (df = 3), *p* = 0.001, *V* = 0.267	10 (7.7)	6 (5.6)	χ^2^ = 4.834 (df = 3), *p* = 0.184
18.5-24.9	131	78 (65.5)	53 (44.5)		74 (56.9)	57 (52.8)	
25-29.9	79	31 (26.1)	48 (40.3)		43 (33.1)	36 (33.3)	
≥30	12	1 (0.8)	11 (9.2)		3 (2.3)	9 (8.3)	
Adherence score (mean during first year after tx)	240						
<87%	110	42 (35.0)	68 (56.7)	χ^2^ = 11.345 (df = 1), *p* = 0.001, *V* = −0.217	60 (45.5)	50 (46.3)	χ^2^ = 0.017 (df = 1), *p* = 0.896
≥87%	130	78 (65.0)	52 (43.3)		72 (54.5)	58 (53.7)	
Adherence subscale scores (mean during first year after tx), median (range)	240						
Health perception	240	0 (0-1)	0.2 (0-1.3)	*Z* = −3.102, *p* = 0.002	0.1 (0-1)	0.2 (0-1.3)	*Z* = −2.042, *p* = 0.041
Home spirometry frequency	240	0 (0-1.4)	0 (0-1.7)	*Z* = −1.297, *p* = 0.195	0 (0-1.4)	0 (0-1.7)	*Z* = −0.099, *p* = 0.921
Contact	240	0 (0-0.7)	0 (0-0.9)	*Z* = −2.490, *p* = 0.013	0 (0-0.9)	0 (0-0.8)	*Z* = −0.088, *p* =0.930
Nutrition, exercise	240	0.3 (0-1.7)	0.3 (0-1.7)	*Z* = −1.322, *p* = 0.186	0.3 (0-1.7)	0.3 (0-1.7)	*Z* = −1.009, *p* = 0.313
Trough levels	240	0.6 (0-5)	0.6 (0-1.4)	*Z* = −0.862, *p* = 0.389	0.6 (0-1.4)	0.7 (0-2)	*Z* = −0.252, *p* = 0.801
Total score	240	1 (0-5)	1.4 (0-5.7)	*Z* = −1.869, *p* = 0.062	1.3 (0-5.4)	1.2 (0.2-5.7)	*Z* = −0.719, *p* = 0.472
Percentage	240	90 (50-100)	86 (43.3-100)	*Z* = −1.869, *p* = 0.062	87.5 (45.7-100)	88 (43.3-98.3)	*Z* = −0.719, *p* = 0.472
Qol VAS, median (range)	235	8 (1-10)	8 (2-10)	*Z* = −0.275, *p* = 0.784	8 (2-10)	8 (1-10)	*Z* = −0.176, *p* = 0.860
PHQ 4 total (0-12), median (range)	235	0 (0-8)	0 (0-12)	*Z* = −0.240, *p* = 0.911	0 (0-5)	0 (0-12)	*Z* = −2.477, *p* = 0.013
PHQ 4 median split	235						
<1	143	74 (62.7)	69 (59.0)	χ^2^ = 0.344 (df = 1), *p* = 0.557	89 (67.9)	54 (51.9)	χ^2^ = 6.242 (df = 1), *p* = 0.012, V = 0.163
≥1	92	44 (37.3)	48 (41.0)		42 (32.1)	50 (48.1)	

*Univariate analyses (Mann-Whitney tests, Chi square tests).*

*BMI, Body Mass Index; CCI, Charlson Comorbidity Index; eGFR, estimated glomerular filtration rate; FEV1, forced expiratory volume in 1 second; PHQ-4, Patient Health Questionnaire-ultrashort version; QoL VAS, quality of life visual analog scale; TERS, Transplant Evaluation Rating Scale; tx, transplantation; V, Cramer V (effect size)*.

In patients with extended 1-year assessment, we also conducted a BAASIS interview. Even though the difference between TERS tertiles concerning adherent and non-adherent patients according to the BAASIS did not reach statistical significance, the effect size (Cramer-V = 0.189) was comparable to the effect size found for the differences with regard to our comprehensive adherence assessment (Cramer-V = 0.175) (Table 6).

**Table 6:** Comparison of extended 1-year post-transplant outcomes according to TERS tertiles (*n* = 143).

**Variable**	***N***	**TERS ≤ 28**	**TERS 29-31,5**	**TERS ≥ 32**	****χ^2^ or *H*,****
		***N* = 80**	***N* = 81**	***N* =79**	***p*-value, effect size**
**TERS scores**, ***n*****(%) or median (range)**					
BAASIS at 1 year	143				
Adherent	125	38 (86.4)	45 (95.7)	42 (80.8)	χ^2^ = 5.095 (df = 2), *p* = 0.078, V = 0.189
Not adherent	18	6 (13.6)	2 (4.3)	10 (19.2)	
SF-8 PCS, median (range)	136	55.7 (20.3-61.1)	52.4 (23.7-61.8)	51 (25.5-58.6)	*H* = 4.358, *p* = 0.113
SF8 MCS, median (range)	136	57.5 (35-62.8)	57.2 (30.8-61.9)	57.2 (23.2-66.9)	*H* = 0.775, *p* = 0.679
PHQ 9 total, median (range)	137	1 (0-12)	3 (0-16)	4 (0-16)	H=7.674, p=.022, η^2^=.042
PHQ-9 cutoffs	137				
0-4	99	35 (81.4)	35 (77.8)	29 (59.2)	χ^2^ = 8.159 (df = 6), *p* = 0.227
5-9 (mild)	30	6 (14.0)	8 (17.8)	16 (32.7)	
10-14 (moderate)	5	2 (4.7)	1 (2.2)	2 (4.1)	
15-27 (severe)	3	0 (0)	1 (2.2)	2 (4.1)	
GAD 7 total, median (range)	136	0 (0-19)	1 (0-16)	1 (0-16)	*H* = 2.868, *p* = 0.238
GAD 7 cutoffs	136				
0-4	116	39 (90.7)	37 (82.2)	40 (83.3)	χ^2^ = 5.658 (df = 6), *p* = 0.463
5-9 (mild)	16	4 (9.3)	7 (15.6)	5 (10.4)	
10-14 (moderate)	2	0	0	2 (4.2)	
15-21 (severe)	2	0	1 (2.2)	1 (2.1)	
PQLS total, median (range)*	114	34 (25-74)	40.5 (25-82)	50 (26-83)	*H* = 12.018, *p* = 0.002, η^2^ = 0.09
Task interference	115	11.2 (8-28)	14 (8-28)	20 (8-38)	*H* = 14.040, *p* = 0.001, η^2^ = 0.108
Psychological functioning	127	9 (7-21)	10 (7-35)	9 (7-25)	*H* = 0.448, *p* = 0.799
Physical functioning	134	4 (4-18)	5 (4-20)	7 (4-20)	*H* = 12.621, *p* = 0.002 η^2^ = 0.081

*Univariate analyses (Kruskal-Wallis H-tests, Chi square tests).*

*BAASIS, Basel Assessment of Adherence to Immunosuppressive Medication Scale; GAD-7, Generalized Anxiety Disorder Screener; PHQ-9, Patient Health Questionnaire-Depression; PQLS, Pulmonary-Specific Quality-of-Life Scale; SF-8 MCS, Short Form 8 Mental Component Summary; SF-8 PCS, Short Form 8 Physical Component Summary; TERS, Transplant Evaluation Rating Scale.*

*Part of the PQLS data were published previously in manuscripts assessing the validity of the TERS and of the German version of the PQLS (21, 37).

#### Psychological Outcomes

One year after transplantation, only two patients exhibited a PHQ-4 score of 6 or above which is considered indicative for the presence of a depressive or anxiety disorder. Four patients scored 3 or above in the two-item depression subscale and two patients in the two-item anxiety subscale. 143 patients (60%) did not report any symptoms on the PHQ-4 with a total score of 0. PHQ-4 did not differ between TERS groups (Table 4); however, there were differences in the “emotional sensitivity” subscale, with patients with scores above the median exhibiting higher PHQ-4 scores (Table 5). This was confirmed by a logistic regression analysis controlling for baseline variables (Supplementary Table 3).

The QoL VAS exhibited median values around 8 and did not differ between TERS groups (Table 4).

### Subsample With Extended 1-Year Assessment

143 patients received a more detailed assessment including the BAASIS interview (see above) and were asked to complete additional questionnaires including the PHQ-9, GAD-7, SF-8, and PQLS to complement the minimal psychosocial assessment with the PHQ-4 and the QoL VAS that are routinely completed by all patients.

This subgroup was fairly evenly distributed between the three TERS tertiles, with 44, 47, and 53 patients, respectively. Importantly, this subsample did not differ from the entire sample of 240 patients who completed the 1-year follow-up in any of the baseline data or outcomes (Table 1); thus, the subsample was most likely representative of the entire follow-up sample.

We found differences in PHQ-9 and GAD-7 scores between the TERS tertiles which were mainly based on differences in the “emotional sensitivity” subscale. The average levels of depression and anxiety tended to be low, with very few patients reporting scores of 10 points or above. A clear association of the TERS tertiles was found with the lung specific quality of life scale PQLS which was predicted by both subscales (Tables 6, 7).

**Table 7:** Comparison of extended 1-year follow-up outcomes divided by the TERS subscales “defiance” and “emotional sensitivity” (median split).

**Variable**	***N***	**Defiance** **≤ 18.75,** ***N* = 120**	**Defiance** **> 18.75,** ***N* = 120**	****χ^2^ or *Z*, *p*-value, effect size****	**Emotional** **≤ 10,** ***N* = 132**	**Emotional** **> 10,** ***N* = 108**	**χ^2^ or *Z*, *p*-value, effect size**
**TERS subscales median split;** ***n*** **(%) or median (range)**							
BAASIS at one year	143						
Adherent	125	62 (91.2)	63 (84.0)	1.669 (df = 1), *p* = 0.196, *V* = 0.108	65 (86.7)	60 (88.2)	0.080 (df = 1), *p* = 0.778
Not adherent	18	6 (8.8)	12 (16.0)		10 (13.3)	8 (11.8)	
SF-8 PCS, median (range)	136	53.5 (20.3-61.8)	52.4 (25.5-58.8)	*Z* = −1.519, *p* = 0.129	54.4 (20.3-61.1)	51.9 (23.4-61.8)	*Z* = −1.229, *p* = 0.219
SF-8 MCS, median (range)	136	56.7 (30.8-62.8)	57.4 (23.3-66.9)	*Z* = −0.508, *p* = 0.612	57.5 (28.9-62.8)	57.0 (66.9-43.7)	*Z* = −1.412, *p* = 0.158
PHQ 9 total, median (range)	137	2 (0-16)	3 (0-16)	*Z* = −0.839, *p* = 0.402	2 (0-13.5)	3.5 (0-16)	*Z* = −3.119, *p* = 0.002 η^2^ = 0.069
PHQ-9 cutoff	137						
0-4	99	50 (75.8)	49 (69.0)	χ^2^ = 1.563 (df = 3),*p* = 0.668, *V* = 0.107	59 (80.8)	40 (62.5)	χ^2^ = 8.425 (df = 3), *p* = 0.038, *V* = 0.248
5-9 (mild)	30	12 (18.2)	18 (25.4)		11 (15.1)	19 (29.7)	
10-14 (moderate)	5	3 (4.5)	2 (2.8)		3 (4.1)	2 (3.1)	
15-27 (severe)	3	1 (1.5)	2 (2.8)		0 (0)	3 (4.7)	
GAD 7 total, median (range)	136	1 (0-16)	1 (0-16)	*Z* = −0.236, *p* = 0.814	0 (0-11)	1 (0-16)	*Z* = −2.157, *p* = 0.031 η^2^ = 0.03
GAD 7 cutoff	136						
0-4	116	54 (81.8)	62 (88.6)	χ^2^ = 4.688 (df = 3),*p* = 0.196, *V* = 0.186	66 (90.4)	50 (79.4)	χ^2^ = 4.496 (df = 3), *p* = 0.213, *V* = 0.182
5-9 (mild)	16	11 (16.7)	5 (7.1)		6 (8.2)	10 (15.9)	
10-14 (moderate)	2	0 (0)	2 (2.9)		1 (1.4)	1 (1.6)	
15-21 (severe)	2	1 (1.5)	1 (1.4)		0 (0)	2 (3.2)	
PQLS total, median (range)	114	38 (25.0-80.4)	46 (25.0-83.0)	*Z* = −2.276 *p* = 0.023η^2^ = 0.045	37 (25-83)	48 (26-80.4)	*Z* = −3.136, *p* = 0.002 η^2^ = 0.086
Task interference	115	14 (8-33)	17.6 (8-38)	*Z* = −2.026, *p* = 0.043η^2^ = 0.035	12.5 (8-30)	18 (8-38)	*Z* = −3.467. *p* = 0.001 η^2^ = 0.104
Psychological functioning	127	9 (7-22)	9 (7-35)	*Z* = −0.816, *p* = 0.415	8.6 (7-35)	9.3 (7-25)	*Z* = −1.037, *p* = 0.300
Physical functioning	134	4 (4-20)	7 (4-20)	*Z* = −3.554, *p* <0.001η^2^ = 0.09	5 (4-20)	6 (4-20)	*Z* = −1.228, *p* = 0.219

*Univariate analyses (Mann-Whitney tests, Chi square tests).*

*BAASIS, Basel Assessment of Adherence to Immunosuppressive Medication Scale; GAD-7, Generalized Anxiety Disorder Screener; PHQ-9, Patient Health Questionnaire-Depression; PQLS, Pulmonary-Specific Quality-of-Life Scale; SF-8 MCS, Short Form 8 Mental Component Summary; SF-8 PCS, Short Form 8 Physical Component Summary; TERS, Transplant Evaluation Rating Scale; V, Cramer V (effect size)*.

The SF-8 composite summary scores did not differ between TERS tertiles and were almost identical to reference values from the German general population (39). The median of the PCS for the entire sample (*n* = 136) was 52.8 (German population 53.6) and the median of the MCS was 57.2 (German population 57.3) (Tables 6, 7).

## Discussion

In this large prospective analysis it could be demonstrated that psychosocial factors as measured by TERS score are predictive of 1-year transplantation outcomes. Patients with lower psychosocial risk were more likely to be listed. The TERS was predictive of behavioral outcomes such as the BMI, adherence, and psychological outcomes such as levels of depression and anxiety, and lung-specific quality of life at 1-year follow-up. The TERS subscales “defiance” and “emotional sensitivity” showed differential predictive validity. While the “defiance” scale score was associated with behavioral outcomes, the “emotional sensitivity” subscale score was predictive for psychological outcomes. Thus, our results support the assumption put forward by Hoodin and Kalbleisch (20) that the TERS is actually a multifaceted construct composed of two subordinate constructs. While related to each other empirically and logically, the two subscales can and should be distinguished conceptually and measured separately.

### Medical Outcomes

Even though the prediction of mortality during the first year after transplantation approached significance, this result should not be over interpreted. Mortality rate during the first year after transplantation was low with 20 patients (7.4%) and chronic lung allograft dysfunction is generally rare during the first year. The few studies that reported the association of TERS scores with mortality and graft functioning included markedly longer follow up periods (10, 16). In longer follow-up examinations, mortality should be used as a time dependent variable instead as a binary outcome. Some patients with high-risk TERS scores who were considered unfit for transplantation may not have been offered transplantation. Differences in TERS scores between listed and not listed patients support this. Unfortunately, the data for such patients are not captured in our database.

None of the other medical outcomes were predicted by TERS tertile scores. Most likely, medical issues during the first year after transplantation are predominantly influenced by the transplant process itself rather than by psychosocial issues. This might change, however, in the long run.

### Behavioral Outcomes

Non-adherence to the medical regimen after transplantation can contribute to poor clinical outcomes (26, 41, 42). Adherence is not only important regarding medication-taking but after lung transplantation also with regard to the regular use of spirometry and other clinical care requirements, regular visits to the transplant center, and lifestyle activities such as nutrition and exercise. Thus, we used a composite adherence measure that has shown to predict mortality and graft loss in a large sample of lung transplant patients (26). The TERS, specifically the “defiance” subscale, was predictive of suboptimal adherence during the first post-transplant year in our sample. Looking at the individual components of non-adherence, especially health perception and regular contacts with the transplant center were predicted by the TERS but not home spirometry frequency, nutrition and exercise or trough levels. Looking at the BAASIS, which was used as an interview and focuses exclusively on adherence to immunosuppressive medication during the last 4 weeks, we found no differences between TERS groups. However, the non-adherence rate was low (12.6%) and the BAASIS covers only a short time period of 4 weeks. Additionally, it has to be kept in mind that suboptimal adherence increases with increasing time since transplantation. In a large sample of lung transplant patients Drick et al. (27) could demonstrate that 37% of all non-adherent patients were transplanted ≥8 years prior to BAASIS assessment. Thus, during the first year adherence is usually higher and will most likely decline with time, which has also been shown in other solid organ transplantation samples (42). The predictive ability of the TERS might be stronger in the longer term after transplantation.

While no association was found between BMI category and TERS tertiles pre-surgery, TERS predicted BMI category at 1-year. It is well-known that obesity is an independent risk factor for mortality and transplant failure after lung transplantation (43). A systematic review and meta-analysis (44) clearly demonstrated that among post-lung transplant recipients underweight and obesity before transplantation were significantly associated with higher mortality and that obesity and overweight were associated with a higher risk of primary graft dysfunction compared to recipients who have normal BMI. A large US registry study including >17,000 patients, confirmed these results and additionally found that BMI increase and decrease from a baseline BMI with the lowest probability of death incrementally increased the odds of mortality at 90 days and 1 year after transplantation (45). The mechanisms are not entirely clear; however, *via* mechanical and probably metabolic effects, lung mechanics are altered in the presence of obesity (46). Weight loss before transplantation was associated with improved short- and long-term clinical outcomes, independent of initial weight (47), and a first case reports describes the successful bariatric surgery in a young women with a BMI of 53.6 kg/m^2^ 4 years after lung transplantation (48).

Taken together, successfully predicting behavioral outcomes such as adherence to a broad range of medical regimens and unfavorable weight developments might be pivotal for mortality and morbidity in lung transplant patients.

### Psychological Outcomes

Even though survival is the key outcome, patients' post-transplant quality of life has become an important component of any evaluation of benefits, specifically as survival times increase (22, 49–52).

As shown in our study, higher levels of pre-transplant “emotional sensitivity” scores might be predictive of lower pulmonary-specific quality of life after transplantation. The PQLS total and subscale values were comparable to the values from the original validation study of the PQLS that provided data at 6 months after transplantation (25). HRQoL was also measured with a generic instrument, the SF-8. TERS tertiles were not predictive of SF-8 subscales; however, in line with other studies in transplant populations, the two subscales—PCS and MCS—have reached values that were comparable to the reference values of the German general population despite differences in life expectancy, treatment-related side effects, and despite the fact that patients after lung transplantation have persistent disabilities (50, 52). Specifically during the first year after transplantation patients experience a substantial benefit from the transplant procedure. Longer-term follow-up will show if we will discover a HRQoL decline after the first post-operative year also in our sample and if this decline is predicted only by co-morbid medical conditions or also by pre-transplant TERS scores.

Comparable to HRQoL measures, depression and anxiety scores were quite low 1 year after transplantation. Again, higher levels of pre-transplant “emotional sensitivity” scores were predictive of higher depression and anxiety scores after transplantation. Overall, predicting post-transplant symptoms of depression might be more important than the presence of pre-transplant mental comorbidity. A meta-analysis on the effect of pre-transplant depression and anxiety on survival following lung transplant (53) did not find that depression or anxiety scores pre-transplant were associated with worse survival. Thus, the presence of affective or anxiety symptoms in a prospective candidate should not be the basis of exclusion from consideration for lung transplantation. However, others found that pre-transplant depression might be a predictor of survival in subgroups of patients (12) and that specifically persistent depression (11) and early post-transplant depressive symptoms might be predictors of long-term outcomes compared with pre-transplant psychosocial assessment alone (7). Smith et al. (7) reported that higher levels of depression and general distress measured 6 months following lung transplantation were associated with increased mortality, independent of baseline characteristics and medical predictors. Also others confirmed that early post-transplant depressive symptoms increase the risk for long-term transplant-related morbidity and mortality (54). Thus, attention should be paid to post-transplant depressive symptoms and putative predictors of the development. Finally, if treatment of comorbid mental disorders would reduce post-transplant mortality requires further study (55).

New psychosocial assessment tools such as the Stanford Integrated Psychosocial Assessment for Transplantation (SIPAT) (56) have been developed and are increasingly used internationally. The SIPAT comprises 18 psychosocial risk factors, which are divided into four domains. The SIPAT has shown to have good interrater reliability (0.85) and to be predictive of medical and psychosocial post-transplant outcomes in a mixed group of organ transplant recipients (57) including rejection episodes, medical hospitalization, infection rates, psychiatric decompensation, and support system failure. They also reported a trend concerning the relationship with non-adherence. As in our study, effect sizes were small to moderate.

### Limitations

Our data are based on a relatively modest sized cohort from a single center with follow-up so far only over 1 year. While our study is the largest to focus on the predictive value of the TERS on multiple post-transplant outcomes, its limitations in size and duration nonetheless are relevant. It has to be kept in mind that patients who get listed and transplanted represent a selected population since transplant centers have to concur with the guidelines of the German Medical Association, which includes the LAS score. In Germany, we also follow the recommendations of International Society for Heart and Lung Transplantation (ISHLT) (58). This must be considered when comparing studies. Psychosocially, our sample was fairly healthy, with overall low TERS values and low mortality rates as well as low levels of depression and anxiety at baseline and at follow-up. Thus, distribution problems caused by considerable ceiling and floor effects, respectively, prevail. Nevertheless, we found significant associations between the pre-transplant TERS and several post-transplant outcomes.

Quantiles are frequently used to facilitate communication of the results to the public and other scientists. Even though the use of quantiles (in our study tertiles) remains highly common in epidemiological research, important problems arise when continuous variables (TERS scores) are categorized, particularly if data dependent cut points are used to form categories (59). Additionally, categorization involves multiple hypothesis testing and assumes homogeneity of risk within groups.

### Conclusion

Our results confirm and extend prior evidence suggesting that psychosocial factors as measured with the TERS may predict medical, behavioral and psychological outcomes following lung transplantation, even during the first post-transplant year.

These findings can have several consequences: Higher psychosocial risk might (1) contribute to the determination of transplant candidacy, (2) lead to interventions prior to listing to reduce or minimize psychosocial risk (e.g., achieve smoking cessation, stabilize mood disorder, strengthen support system), and/or (3) might lead to increased clinical attention (“red flags”) throughout the transplantation process and guide proactive interventions. Transplant physicians and mental health professionals should discuss the interventions required to be able to safely offer transplantation (10, 16) and the behavioral interventions necessary to avoid or minimize behavioral complications. Longer-term prospective follow-ups are needed since during the first post-transplant year the predictive impact of psychosocial risk factors might differ from that during consecutive years.

## Data Availability Statement

The datasets generated for this study are available on request to the corresponding author.

## Ethics Statement

The studies involving human participants were reviewed and approved by Institutional Ethics Board of Hannover Medical School. The patients/participants provided their written informed consent to participate in this study.

## Author Contributions

MZ and JG designed the study. MZ and MB-H were mainly responsible for data acquisition. MZ, CV, and MN analyzed the data. MZ and MN wrote the first draft. All authors contributed significantly to the interpretation of the data and the final version of the manuscript and gave final approval of the version to be published.

## Conflict of Interest

The authors declare that the research was conducted in the absence of any commercial or financial relationships that could be construed as a potential conflict of interest.

## Publisher's Note

All claims expressed in this article are solely those of the authors and do not necessarily represent those of their affiliated organizations, or those of the publisher, the editors and the reviewers. Any product that may be evaluated in this article, or claim that may be made by its manufacturer, is not guaranteed or endorsed by the publisher.
