# Supplementary material for: The Transplant Evaluation Rating Scale Predicts Clinical Outcomes 1 Year After Lung Transplantation: A Prospective Longitudinal Study
Source: Front Psychiatry. 2021 Aug 26;12:704319. doi: 10.3389/fpsyt.2021.704319 (PMC8426579; doi:10.3389/fpsyt.2021.704319)
Supplement: Supplementary file 1 [file Data_Sheet_1.docx]

**Supplementary Table 1**: BInary logistic regression models predicting good adherence (composite adherence score ≥87%)

1. TERS tertiles

|  | N | Odds Ratio | 95% Confidence Interval | p-value |
| --- | --- | --- | --- | --- |
| TERS tertiles |  |  |  | .042 |
| Tertile 3 (≥32) | 79 | (Ref) | (Ref) | (Ref) |
| Tertile 1 (≤28) | 80 | 2.07 | 1.05-1.09 | .035 |
| Tertile 2 (29-31.5) | 80 | 2.07 | 1.09-3.94 | .026 |
| Sex |  |  |  |  |
| Female | 114 | (Ref) | (Ref) | (Ref) |
| Male | 125 | 0.85 | 0.49-1.48 | .571 |
| Age | 239 | 0.99 | 0.96-1.02 | .368 |
| Educational level |  |  |  |  |
| <12 years | 165 | (Ref) | (Ref) | (Ref) |
| ≥12 years | 74 | 0.91 | 0.50-1.66 | .762 |
| LAS Category |  |  |  | .891 |
| D (restrictive) | 101 | (Ref) | (Ref) | (Ref) |
| A (obstructive) | 79 | 0.83 | 0.43-1.60 | .580 |
| B (circulation) | 14 | 0.66 | 0.20-2.19 | .497 |
| C (infectious, CF) | 45 | 0.90 | 0.35-2.33 | .835 |

N=239, Hosmer-Lemeshow-Test: Χ^2^=8.87 (df=8), p=.354

CF, cystic fibrosis

1. Defiance

|  | N | Odds Ratio | 95% Confidence Interval | p-value |
| --- | --- | --- | --- | --- |
| Defiance |  |  |  |  |
| Above median split (>18.75) | 120 | (Ref) | (Ref) | (Ref) |
| Below median split (≤18.75) | 119 | 2.58 | 1.43-4.68 | .002 |
| Sex |  |  |  |  |
| Female | 114 | (Ref) | (Ref) | (Ref) |
| Male | 125 | 0.91 | 0.52-1.59 | .738 |
| Age | 239 | 0.99 | 0.96-1.02 | .417 |
| Educational level |  |  |  |  |
| <12 years | 165 | (Ref) | (Ref) | (Ref) |
| ≥12 years | 74 | 0.82 | 0.45-1.51 |  |
| LAS Category |  |  |  | .907 |
| D (restrictive) | 101 | (Ref) | (Ref) | (Ref) |
| A (obstructive) | 79 | 1.04 | 0.54-2.01 | .907 |
| B (circulation) | 14 | 0.65 | 0.19-2.18 | .481 |
| C (infectious, CF) | 45 | 0.85 | 0.33-2.20 | .734 |

N=239, Hosmer-Lemeshow-Test: Χ^2^=6.35 (df=8), p=.608

1. Emotional Sensitivity

|  | N | Odds Ratio | 95% Confidence Interval | p-value |
| --- | --- | --- | --- | --- |
| Emotional Sensitivity |  |  |  |  |
| Above median split (>10) | 107 | (Ref) | (Ref) | (Ref) |
| Below median split (≤10) | 132 | 1.02 | 0.61-1.72 | .936 |
| Sex |  |  |  |  |
| Female | 114 | (Ref) | (Ref) | (Ref) |
| Male | 125 | 0.88 | 0.51-1.52 | .640 |
| Age | 239 | 0.99 | 0.96-1.02 | .335 |
| Educational level |  |  |  |  |
| <12 years | 165 | (Ref) | (Ref) | (Ref) |
| ≥12 years | 74 | 1.01 | 0.57-1.81 | .970 |
| LAS Category |  |  |  | .798 |
| D (restrictive) | 101 | (Ref) | (Ref) | (Ref) |
| A (obstructive) | 79 | 0.76 | 0.41-1.44 | .420 |
| B (circulation) | 14 | 0.65 | 0.20-2.12 | .471 |
| C (infectious, CF) | 45 | 0.96 | 0.38-2.43 | .925 |

N=239, Hosmer-Lemeshow-Test: Χ^2^=18.694 (df=8), p=.017

**Supplementary Table 2:** Logistic regression models predicting overweight and obesity

1. TERS tertiles

|  | N | Odds Ratio | 95% Confidence Interval | p-value |
| --- | --- | --- | --- | --- |
| TERS tertiles |  |  |  | **.079** |
| Tertile 3 (≥32) | 79 | **(Ref)** | **(Ref)** | **(Ref)** |
| Tertile 1 (≤28) | 79 | **0.47** | **0.23-0.99** | **.046** |
| Tertile 2 (29-31.5) | 79 | **0.54** | **0.27-1.06** | **.074** |
| Sex |  |  |  |  |
| Female | 113 | (Ref) | (Ref) | (Ref) |
| Male | 124 | 2.25 | 1.22-4.17 | .010 |
| Age | 237 | 1.02 | 0.98-1.05 | .364 |
| Educational level |  |  |  |  |
| <12 years | 164 | (Ref) | (Ref) | (Ref) |
| ≥12 years | 73 | 0.69 | 0.35-1.36 | .284 |
| LAS Category |  |  |  | .041 |
| D (restrictive) | 101 | (Ref) | (Ref) | (Ref) |
| A (obstructive) | 79 | 1.28 | 0.65-2.54 | .464 |
| B (circulation) | 14 | 2.17 | 0.63-7.51 | .220 |
| C (infectious, CF) | 43 | 0.25 | 0.07-0.91 | .036 |

N=237, Hosmer-Lemeshow-Test: Χ^2^=8.74 (df=8), p=.364

BMI was dichotomized in BMI < 25 and ≥ 25 kg/m^2^

CF, cystic fibrosis

1. Defiance

|  | N | Odds Ratio | 95% Confidence Interval | p-value |
| --- | --- | --- | --- | --- |
| Defiance |  |  |  |  |
| Above median split (>18.75) | 119 | (Ref) | (Ref) | (Ref) |
| Below median split (≤18.75) | 118 | 0.50 | 0.27-0.92 | .027 |
| Sex |  |  |  |  |
| Female | 113 | (Ref) | (Ref) | (Ref) |
| Male | 124 | 2.13 | 1.16-3.92 | .015 |
| Age | 237 | 1.01 | 0.98-1.05 | .410 |
| Educational level |  |  |  |  |
| <12 years | 164 | (Ref) | (Ref) | (Ref) |
| ≥12 years | 73 | 0.72 | 0.37-1.41 | .333 |
| LAS Category |  |  |  | .065 |
| D (restrictive) | 101 | (Ref) | (Ref) | (Ref) |
| A (obstructive) | 79 | 1.14 | 0.58-2.24 | .705 |
| B (circulation) | 14 | 2.22 | 0.64-7.74 | .209 |
| C (infectious, CF) | 43 | 0.27 | 0.07-0.99 | .048 |

N=237, Hosmer-Lemeshow-Test: Χ^2^=7.91 (df=8), p=.443

1. Emotional Sensitivity

|  | N | Odds Ratio | 95% Confidence Interval | p-value |
| --- | --- | --- | --- | --- |
| Emotional Sensitivity |  |  |  |  |
| Above median split (>10.00) | 107 | (Ref) | (Ref) | (Ref) |
| Below median split | 130 | 0.68 | 0.38-1.21 | .185 |
| Sex |  |  |  |  |
| Female | 113 | (Ref) | (Ref) | (Ref) |
| Male | 124 | 2.22 | 1.21-4.10 | .011 |
| Age | 237 | 1.02 | 0.98-1.05 | .328 |
| Educational level |  |  |  |  |
| <12 years | 164 | (Ref) | (Ref) | (Ref) |
| ≥12 years | 73 | 0.65 | 0.33-1.25 | .192 |
| LAS Category |  |  |  | .026 |
| D (restrictive) | 101 | (Ref) | (Ref) | (Ref) |
| A (obstructive) | 79 | 1.39 | 0.73-2.64 | .321 |
| B (circulation) | 14 | 2.35 | 0.68-8.11 | .178 |
| C (infectious, CF) | 43 | 0.25 | 0.07-0.89 | .032 |

N=237, Hosmer-Lemeshow-Test: Χ^2^=5.943 (df=8), p=.654

**Supplementary Table 3**: Logistic regression models predicting PHQ-4 ≥1

1. TERS tertiles

|  | N | Odds Ratio | 95% Confidence Interval | p-value |
| --- | --- | --- | --- | --- |
| TERS tertiles |  |  |  | .300 |
| Tertile 3 (≥32) | 77 | (Ref) | (Ref) | (Ref) |
| Tertile 1 (≤28) | 79 | 0.58 | 0.29-1.17 | .127 |
| Tertile 2 (29-31.5) | 78 | 0.73 | 0.38-1.40 | .338 |
| Sex |  |  |  |  |
| Female | 110 | (Ref) | (Ref) | (Ref) |
| Male | 124 | 1.07 | 0.60-1.89 | .825 |
| Age | 234 | 1.01 | 0.98-1.05 | .365 |
| Educational level |  |  |  |  |
| <12 years | 160 | (Ref) | (Ref) | (Ref) |
| ≥12 years | 74 | 0.74 | 0.40-1.38 | .345 |
| LAS Category |  |  |  | .778 |
| D (restrictive) | 98 | (Ref) | (Ref) | (Ref) |
| A (obstructive) | 78 | 1.00 | 0.52-1.95 | .993 |
| B (circulation) | 14 | 1.69 | 0.5-5.70 | .394 |
| C (infectious, CF) | 44 | 0.86 | 0.32-2.34 | .769 |

N=234, Hosmer-Lemeshow-Test: Χ^2^=6.261 (df=8), p=.618

CF, cystic fibrosis

1. Defiance

|  | N | Odds Ratio | 95% Confidence Interval | p-value |
| --- | --- | --- | --- | --- |
| Defiance |  |  |  |  |
| Above median split (>18.75) | 117 | (Ref) | (Ref) | (Ref) |
| Below median split (≤18.75) | 117 | 1.15 | 0.63-2.10 | .645 |
| Sex |  |  |  |  |
| Female | 110 | (Ref) | (Ref) | (Ref) |
| Male | 124 | 1.05 | 0.59-1.85 | .873 |
| Age | 234 | 1.02 | 0.98-1.05 | .334 |
| Educational level |  |  |  |  |
| <12 years | 160 | (Ref) | (Ref) | (Ref) |
| ≥12 years | 74 | 0.67 | 0.36-1.24 | .199 |
| LAS Category |  |  |  | .907 |
| D (restrictive) | 98 | (Ref) | (Ref) | (Ref) |
| A (obstructive) | 78 | 1.16 | 0.60-2.25 | .664 |
| B (circulation) | 14 | 1.77 | 0.53-5.89 | .354 |
| C (infectious, CF) | 44 | 0.83 | 0.31-2.26 | .717 |

N=234, Hosmer-Lemeshow-Test: Χ^2^=6.60 (df=8), p=.580

1. Emotional Sensitivity

|  | N | Odds Ratio | 95% Confidence Interval | p-value |
| --- | --- | --- | --- | --- |
| Emotional Sensitivity |  |  |  |  |
| Above median split (>10) | 103 | (Ref) | (Ref) | (Ref) |
| Below median split (≤10) | 131 | 0.49 | 0.28-0.84 | .010 |
| Sex |  |  |  |  |
| Female | 110 | (Ref) | (Ref) | (Ref) |
| Male | 124 | 1.1 | 0.62-1.96 | .744 |
| Age | 234 | 1.02 | 0.98-1.05 | .307 |
| Educational level |  |  |  |  |
| <12 years | 160 | (Ref) | (Ref) | (Ref) |
| ≥12 years | 74 | 0.71 | 0.38-1.31 | .271 |
| LAS Category |  |  |  | .652 |
| D (restrictive) | 98 | (Ref) | (Ref) | (Ref) |
| A (obstructive) | 78 | 1.03 | 0.54-1.97 | .922 |
| B (circulation) | 14 | 1.90 | 0.56-6.44 | .301 |
| C (infectious, CF) | 44 | 0.82 | 0.30-2.24 | .697 |

N=234, Hosmer-Lemeshow-Test: Χ^2^=7.398 (df=8), p=.494
